# Serum Free Amino Acid Profiling in Differential Diagnosis of Ovarian Tumors—A Comparative Study with Review of the Literature

**DOI:** 10.3390/ijerph18042167

**Published:** 2021-02-23

**Authors:** Agnieszka Horala, Szymon Plewa, Pawel Derezinski, Agnieszka Klupczynska, Jan Matysiak, Ewa Nowak-Markwitz, Zenon J. Kokot

**Affiliations:** 1 Gynecologic Oncology Department, Poznan University of Medical Sciences, Polna 33 Street, 60-535 Poznan, Poland; ewamarkwitz@ump.edu.pl; 2 Department of Inorganic and Analytical Chemistry, Poznan University of Medical Sciences, 6 Grunwaldzka Street, 60-780 Poznan, Poland; szymonplewa1@gmail.com (S.P.); p.derezinski@gmail.com (P.D.); aklupczynska@ump.edu.pl (A.K.); jmatysiak@ump.edu.pl (J.M.); 3 Faculty of Health Sciences, Calisia University, 13 Kaszubska Street, 62-800 Kalisz, Poland; z.kokot@akademiakaliska.edu.pl

**Keywords:** ovarian cancer, ovarian neoplasm, ovarian tumour, biomarker, amino acids, metabolomics, metabolic profiling

## Abstract

Proper preoperative ovarian cancer (OC) diagnosis remains challenging. Serum free amino acid (SFAA) profiles were investigated to identify potential novel biomarkers of OC and assess their performance in ovarian tumor differential diagnosis. Serum samples were divided based on the histopathological result: epithelial OC (*n* = 38), borderline ovarian tumors (*n* = 6), and benign ovarian tumors (BOTs) (*n* = 62). SFAA profiles were evaluated using aTRAQ methodology based on high-performance liquid chromatography electrospray ionization tandem mass spectrometry (HPLC-ESI-MS/MS). Levels of eleven amino acids significantly differed between OC+borderline and BOTs. The highest area under the receiver operating characteristic curve (AUC of ROC) (0.787) was obtained for histidine. Cystine and histidine were identified as best single markers for early stage OC/BOT and type I OC. For advanced stage OC, seven amino acids differed significantly between the groups and citrulline obtained the best AUC of 0.807. Between type II OC and BOTs, eight amino acids differed significantly and the highest AUC of 0.798 was achieved by histidine and citrulline (AUC of 0.778). Histidine was identified as a potential new biomarker in differential diagnosis of ovarian tumors. Adding histidine to a multimarker panel together with CA125 and HE4 improved the differential diagnosis between OC and BOTs.

## 1. Introduction

The research for elaborating efficient ovarian cancer (OC) diagnostic tools has been ongoing for decades. At present, no screening method is available and the disease has a highly unfavorable prognosis, mainly due to the fact that over 70% of the patients are diagnosed in late stages, i.e., stage III and IV according to the International Federation of Gynaecology and Obstetrics (FIGO). Early and specific diagnosis is essential to improve the treatment outcome, because five year survival rates for FIGO stage I reach 90%, compared to about 30% for advanced disease (FIGO stage III–IV) [1].

One of the challenges in OC diagnosis is the correct differentiation of ovarian tumors noticed on routine transvaginal ultrasound examination. Proper preoperative risk-of-malignancy assessment is very important for making clinical decisions and treatment planning. Low-risk tumors can be followed up and re-assessed by imaging methods after a certain period of time or operated conservatively, ensuring fertility-sparing, and in a less invasive way (e.g., unilateral laparoscopic tumorectomy). In the case of high-risk lesions, additional examinations may be needed preoperatively to adequately plan the surgery and prepare the patient (computed tomography scan, colonoscopy, reservation of appropriate time for surgery, ensuring the availability of intra-operative tissue examination to assess the type of tumor during the surgery, ensuring adequately trained staff, etc.). To date, histological examination of the resected tissue still remains the gold standard. This approach results in unnecessary surgical procedures that, if a reliable non-invasive diagnostic method existed, could be avoided, because over 90% of ovarian masses detected in pre-menopausal women and up to 60% of those in post-menopausal women are benign [2]. Moreover, correct pre-operative diagnosis of OC enables adequate referral of the patient to specialized gynecologic oncology centers where evaluation by an interdisciplinary tumor board and optimal debulking surgery is possible. Treating women with OC in specialized centers is crucial to ensure proper management and was proved to significantly improve the prognosis [3].

Some clinical multivariate diagnostic models used in ovarian tumor differential diagnosis were reported to be quite efficient, for example the ADNEX model (The Assessment of Different NEoplasias in the adneXa), based on ultrasound features and clinical data, was reported to reach an area under the receiver operating characteristic (AUC of ROC) curves as high as 0.954 [4]. However, despite the excellent performance, its clinical application is highly limited due to the need for highly-trained medical staff and modern equipment to perform high-quality ultrasound assessment of ovarian tumors and record the required features for the model. In addition, some ovarian benign tumors pose a particular diagnostic challenge, commonly presenting ultrasound features typical for malignant lesions [5]. For this reason, biomarker research is more likely to provide accessible and ready-to-use diagnostic methods.

Metabolomic profiling has recently become a highly promising target in the search for non-invasive cancer diagnostic methods. Metabolome is defined as a complete set of small molecules within a biological sample. Therefore, it is a direct reflection of the current processes in the organism and is altered by pathological conditions such as carcinogenesis. We have previously showed that the serum free amino acid (SFAA) profiles are altered in ovarian cancer patients [6] and investigated the role of amino acid profiling in screening for OC. The current study was designed to investigate the role of amino acid profiling in preoperative differential diagnosis of ovarian tumors. For this purpose, the SFAA profiles of OC, borderline ovarian tumors, and benign ovarian tumors (BOTs) were analyzed, differentiating amino acids between the groups were selected, and their performance in differential diagnosis of ovarian tumors assessed. To the best of our knowledge, this is the first study of ovarian cancer that analyzes such a wide spectrum of the SFAA profile in differential diagnosis of ovarian tumors.

## 2. Materials and Methods

The study group comprised 122 patients diagnosed with ovarian tumors and qualified for surgical treatment in the Gynecologic Oncology Department between August 2014 and December 2015. The study protocol was approved by the Local Bioethical Committee of Poznan University of Medical Sciences, Poland (decision no. 165/16 of 4 February 2016 and 80/17 of 5 January 2017). A written consent to participate in the study was obtained from all patients prior to sample collection. The blood samples from partially the same cohort (OC and BOT samples) were also used in our previous study [6]. The sample collection and preparation were undertaken following the same protocol as described previously [6].

The exclusion criteria were: any other malignancy currently or in anamnesis, ovarian malignancy other than primary epithelial OC, previous OC treatment, chronic liver diseases, diabetes, chronic renal failure, and malnutrition (defined as weight loss >10% in the past 3 months), and were met by 16 patients. Samples from 106 patients were included in the final analysis and divided based on the histopathological result: OC (*n* = 38), borderline ovarian tumors (*n* = 6) and BOT (*n* = 62). In addition, the OC group was divided into type I OC (*n* = 13; borderline tumors were included in this group) and type II OC (*n* = 31) according to the clinicopathological classification proposed by Kurman [7].

Because our study focused on differential diagnosis of ovarian tumors, borderline tumors were included in all analyses to better reflect the real population and avoid study selection bias. Taking into account the increasing evidence that low-grade serous carcinomas (type I OC) develop from borderline tumors and that the pathways and genes involved in their pathogenesis are distinct from those of high-grade serous carcinomas (type II OC) [7,8], borderline tumors were added to the type I OC group for statistical analyses.

### 2.1. Amino Acid Profiling

A panel composed of 42 amino acids and biogenic amines (Table 1) was quantitatively measured in all 106 samples. Of these, 20 amino acids are encoded in the standard genetic code and are proteinogenic, which means they are used to biosynthesize proteins during translation; all of these amino acids were included into the final analysis. The methodology of determining SFAA was described by us in the previous publication [6].

CA125 and HE4 serum concentrations were quantitatively measured by electrochemiluminescence immunoassay (ECLIA) on Roche Cobas System (Roche Diagnostics, Indianapolis, IN, USA) in the Central Hospital Laboratory according to the manufacturer’s instructions. The standard cut-off values are 35 U/mL for CA125 and 140 pmol/L for HE4, however for the purpose of this study optimal cut-off levels were identified.

### 2.2. Data Analysis

The statistical assessment was carried out using STATISTICA 12.5 (StatSoft Inc., Tulsa, OK, USA) software and MetaboAnalyst web server [9]. Firstly, the normality of distribution of all data sets was tested using the Shapiro–Wilk test. In the case of non-normally distributed variables, the Mann–Whitney U test was applied to evaluate the differences in SFAA between the OC group and the benign ovarian tumor group. Conversely, when the data was normally distributed, Levene’s test was used to evaluate the equality of variances. When variances were equal, the *t*-test was applied for further statistical assessment, otherwise the Welch *t*-test was used. In all statistical tests, *p*-value < 0.05 was regarded as significant. In the second step, the univariate ROC curves were created for each of the analyzed amino acid. Metabolites characterized by the highest AUC in univariate ROC were selected to perform multivariate ROC curve analyses. For this purpose, data normalization by sum, logarithm transformation and auto scaling were carried out. The obtained AUC of ROC curves were compared to assess the discriminatory ability of the models.

## 3. Results

Of 42 analyzed amino acids, 33 were included in the final analysis due to the fact that the concentrations of nine amino acids did not exceed the limit of quantitation (LOQ). There were no statistically significant differences in body mass index (BMI) between the studied groups. Significant differences in age and menopausal status were observed among the groups, as expected in a real-life population taking into account the disease incidence. Detailed study group characteristics are presented in Table 2. All borderline tumors and OC patients underwent complete surgical staging according to the classification by FIGO. Approximately one-third of OC patients had early stage disease (FIGO I-II) (Table 2).

The results of the statistical analyses are presented in Table 3 and Table 4, and Table S1.

### 3.1. Usefulness of Amino Acid Profiling in Differential Diagnosis of Ovarian Tumors: OC vs. BOTs and OC+Borderline Tumors vs. BOTs

In the performed analyses, the levels of ten amino acids significantly differed between OC and BOT (elevated in OC: Aad, Cys, Ile, Leu; decreased in OC: Asn, Cit, Gln, His, Thr, Trp). When borderline tumors were added to the OC group, the level of one additional amino acid (Phe) was significantly increased in the OC/borderline tumors group. The highest AUC in both analyses (0.820 and 0.787, respectively) was obtained by histidine, whose level was significantly reduced in the OC/borderline tumors patients. However, none of the analyzed amino acids obtained an AUC superior to those of CA125 and HE4 (Table 3).

In order to further evaluate the performance of histidine, multivariate models based on two variables (CA125+HE4) and three variables (CA125+HE4+histidine) were created. The analysis revealed that adding histidine to a multi-marker panel improved the diagnostic performance of the test in both analyses (Table 4).

### 3.2. Usefulness of Amino Acid Profiling in Detecting Early Stages of Ovarian Cancer: FIGO I-II (Incl. Borderline Tumors) vs. Benign and FIGO III-IV vs. Benign

To assess the ability of the analyzed markers to detect early stage OC, two subgroups of OC patients (FIGO stage I-II and FIGO stage III-IV) were independently compared against the BOT group. All borderline tumors in our cohort were stage FIGO I and were included in this analysis. Two amino acids (Cys and His) differed significantly between early stage OC and BOT and the highest AUC was achieved by histidine (0.786). Seven amino acids differed significantly between advanced stage OC and BOT (Aad, Cut, Gln, His, Ile, Thr, Trp) and the highest AUC was achieved by citrulline (0.807), although histidine also obtained a high AUC of 0.788. These results confirm that the SFAA profiles become increasingly altered with the progress of OC. Again, none of the analyzed amino acids obtained an AUC superior to those of CA125 and HE4 (Table 3). As expected, the discriminatory ability of CA125 and HE4 was higher for advanced stage OC.

The addition of histidine improved the diagnostic accuracy of a multivariate model for early stage disease (FIGO I-II vs. BOT) and the addition of citrulline improved the diagnostic accuracy of a multivariate model for advanced stage disease (FIGO III-IV vs. BOT), however only a minor increase in the AUC value was observed for the latter (Table 4).

### 3.3. Usefulness of Amino Acid Profiling for Different Ovarian Cancer Types (Type I OC vs. Type II OC, Type I OC vs. Benign, Type II OC vs. Benign)

Having in mind the heterogeneity of OC, this set of analyses was performed to investigate if two clinically and molecularly distinct subtypes of OC (according to Kurman et al. [7]) could possibly have their own specific markers. Borderline tumors were included in this analysis as type I OC. Interestingly, the SFAA profiles of type I and type II OC differed by only one amino acid (citrulline, AUC of 0.730) which suggests that the changes in SFAA profiles are rather common for both types of OC. The levels of histidine, which obtained the highest AUC in almost all other analyses, did not differ significantly between type I and type II OC, which may be suggestive of its universal role in OC diagnosis. In the comparison between type I OC and BOT, two amino acids were differently expressed (Cys and His). It is worth noting that the same amino acids were significantly altered in early stage OC. The fact that type I OC is generally characterized by less aggressive clinical course and thus is more likely to be diagnosed in early stages, corresponds well with these results. When comparing type II OC with BOT, the expression of eight amino acids significantly differed between the groups (Aad, Cit, Gln, His, Ile, Leu, Phe, Trp). The highest discriminatory ability was again achieved by histidine (AUC of 0.798), closely followed by citrulline (AUC of 0.778), which may suggest citrulline as a type II OC marker (particularly because it did not reach statistical significance in type I OC vs. BOT analysis). Additionally, cystine could be distinguished as a potential type I OC marker because it was one of the few amino acids that did reach statistical significance in the type I vs. BOT analysis.

Both CA125 and HE4 obtained high AUC in all analyses and, as expected, their discriminatory ability was higher for type II OC (AUC of 0.965 and 0.972, respectively) than for type I OC (AUC of 0.810 and 0.828, respectively).

In multivariate model analyses, the addition of histidine to a two-marker panel consisting of CA125 and HE4 only slightly raised the respective AUC values (Table 4).

## 4. Discussion

Differential diagnosis of ovarian tumors is an important diagnostic step enabling adequate qualification for surgical management of the patients. Because the ovaries are relatively inaccessible for a preoperative biopsy, which is also contraindicated due to the risk of iatrogenic rupture of the tumor capsule, resulting in the spread of the cancer (“surgical spill”) in the case of malignancy, the markers should ideally be obtainable from an accessible body fluid, such as blood, urine, or saliva. In recent years, thanks to technological advances, metabolomics has emerged as a promising method of searching for new OC biomarkers.

Several studies confirmed that the plasma/serum free amino acids (PFAA/SFAA) profile is significantly altered in cancer patients, e.g., lung, gastric, colorectal, breast, renal, prostate, and endometrial cancers [10,11,12,13,14], and noted that some differences reflected the metabolic changes common to many cancers, whereas others were specific to each type of cancer [10]. In a paper by Miyagi et al. that investigates the PFAA profiles in five types of cancer (lung, gastric, colorectal, breast, prostate), it is suggested that a decrease in glutamine, histidine, and tryptophan and an increase in proline and ornithine might reflect the metabolic changes common to all cancers [10]. Although we indeed observe changes in the levels of glutamine, histidine, and tryptophan, the expression of proline and ornithine was not altered in our cohort. Because the PFAA profile can also differ between the early and late stages of cancer and between subtypes of cancer, we performed a detailed analysis of the PFAA profiles in various clinical subgroups.

Our results identified histidine as the most effective OC marker in almost all analyzed subgroups. Of particular value, its performance did not drop for detection of early stage cancer (AUC of 0.786 and 0.788 for early and late stage OC, respectively). Moreover, it obtained similar results in the comparison between type I or type II OC with BOT and did not differ significantly between the two OC types. Therefore, it could be considered as a universal OC biomarker and should be subject to further research. Histidine was closely followed by tryptophan, which obtained high AUC values, especially in advanced stage and high-grade (type II) OC. The depletion of those two amino acids in OC patients is in line with other studies—see Table 5. Although, as mentioned above, the changes in histidine and tryptophan may be observed in other cancers, this study focused on amino acid profiling for distinguishing benign and malignant ovarian tumors, i.e., the situation in which a pathology in the ovaries is already detected by imaging methods. Nevertheless, the fact that histidine and tryptophan levels are decreased in other cancers is likely to negatively affect their specificity for detecting OC by increasing the number of false positive results in patients whose lesion in the ovary is in fact benign but there is a concomitant malignancy of a different organ.

The results of our study also indicate that citrulline could be considered as a type II OC marker (particularly as it did not reach statistical significance in type I OC vs. BOT analysis). Additionally, cystine could be distinguished as a potential type I OC marker because it was one of the few amino acids that reached statistical significance in the type II vs. BOT analysis. However, the performance of these two amino acids may not be sufficient for clinical use.

Our findings correspond with the results of a paper on high-grade serous OC (equivalent to type II OC in our study) using targeted metabolomics, which reports a decreased serum concentrations of five amino acids (histidine, lysine, threonine, tryptophan, citrulline) compared with healthy controls that also correlated with shorter overall survival of cancer patients [18]. The levels of four of these amino acids (histidine, threonine, tryptophan, citrulline) were decreased in OC patients in our analysis and three (histidine, tryptophan, citrulline) were decreased in type II OC, although it should be noted that the comparison was between OC and BOT (not healthy controls). Other important findings of the above-mentioned study are that the levels of amino acids identified as significant were similar in serum, ascites fluid, and tumor tissue, and that they were positively correlated with the tumor load (i.e., recovered to concentrations typical of healthy patients after initiation of anti-cancer treatment). The authors conclude that this suggests that the depletion of certain amino acids in serum is a direct effect of tumor metabolism [18].

A systematic review of metabolomic studies in OC was published recently [23]. It concluded that the most frequently reported amino acid alterations in OC were the decreased levels of histidine, citrulline, alanine, and methionine [23]. Other studies that analyzed serum samples identified altered levels of several amino acids in OC patients. These results are summarized in Table 5. There are a lot of discrepancies in the amino acids identified as differential and some studies even reveal an opposite trend of a specific metabolite (e.g., alanine, threonine). This might be due to the adoption of different mass spectrometry-based analytic methods to identify those metabolites and different study design, especially regarding control groups. Moreover, all cited studies were based on global rather than targeted metabolomic profiling techniques in which amino acids were only a small proportion of the investigated substances, whereas our study is unique in that it focused purely on SFAA profile. Notwithstanding different study methods, the results are coherent for histidine and tryptophan, which suggests that their levels are strongly affected by OC development.

A study by Hilvo et al. [16] additionally compared the results obtained from serum samples with matching tumor tissue samples and confirmed a linear correlation of diagnostically relevant biomarkers between serum and tumor tissue. These findings support the hypothesis that relevant metabolites originate from the tumor rather than depend on other metabolic processes in the body.

It is not clear, however, the extent to which the studies based on plasma analysis can be compared with our research in which serum samples were collected. Serum is the liquid fraction of whole blood obtained after the blood is allowed to clot and centrifuged. Plasma is obtained when whole blood is collected in tubes treated with an anticoagulant and then centrifuged to remove blood cells. Surprisingly, perhaps, a study comparing amino acid profiles in both types of blood samples revealed remarkable differences in the PFAA and SFAA profiles [12]. In general, the amino acid concentrations were, on average, 40% lower in plasma than in serum, although the level of variation and the direction of changes varied for each individual amino acid. Nevertheless, significant differences were observed in both profiles (SFAA and PFAA) between cancer patients (clear cell renal cancer) and healthy controls, and in serum a decreased level of histidine—the same as in our study—was identified as the most effective cancer marker [12].

In addition to the SFAA profile, in our research two clinically used OC biomarkers, CA125 and HE4, were additionally analyzed. Their generally high performance in differential diagnosis of ovarian tumors was also confirmed by our analyses. As expected, their diagnostic accuracy was lower in detecting early stage and type I OC. Although all of the analyzed amino acids failed to reach a higher AUC than CA125 and HE4, the diagnostic performance of histidine was not subject to OC stage and type. Moreover, the addition of histidine improved the diagnostic performance of all presented multivariate models based on CA125 and HE4.

Most of the amino acids identified in our research as statistically significant were proved to be involved in metabolic pathways altered during cancer growth and progression. Tryptophan depletion triggers apoptosis of effector T cells contributing to the suppression of antitumor immune responses [24]. Considerable evidence indicates that histamine, a derivative of amino acid histidine, may be a crucial mediator in cancer growth and progression by regulating processes such as angiogenesis, cell invasion, migration, differentiation, apoptosis, and modulation of immune responses [25]. Histidine decarboxylase that converts histidine to histamine was found to be overexpressed in several cancers, including OC tissue [26]. Glutamine is used by tumors for nucleotide biosynthesis whereas glutamate, its derivative, serves as a donor of nitrogen for the production of other amino acids. Glutaminase, an enzyme which converts glutamine to glutamate, was found to be frequently upregulated in cancer cells [24].

Among the limitations of this study are the number of patients and the fact that they were all from a single institute. Nonetheless, this ensured the consistency in gathering and processing the samples. The distribution of histological types of OC consisted of serous (42%), endometrioid (11%), clear cell (8%), mucinous (3%), and undifferentiated carcinomas (26%), and the frequency of the last type was much higher than reported in other European countries. This is probably due to an individual bias of the pathology department and some of these cancers could probably have been classified as high-grade serous. In four cases (10%), the type was not identified because the patients were qualified to neoadjuvant chemotherapy and the diagnosis was obtained after a paracentesis of ascites. The study also excluded cancers other than epithelial OC (i.e., germ-cell and stromal cancers). However, taking into account their extremely low incidence (less than 2% of cancers) this factor has very limited clinical impact. Another potential weakness of the presented research is the possibility of a relationship between behavioral and/or dietary patterns of the patients and alterations in the amino acid profiles [27]. To reduce this potential bias, malnourished patients were excluded from the analysis and the blood samples were collected after overnight fasting.

The number of patients in the subgroup analyses (early vs. late stage; type I vs. type II) was especially limited, therefore much larger cohorts are needed to verify the utility of the amino acids indicated in these subgroups as relevant. Nevertheless, since only diagnosis at an early, asymptomatic stage is likely to have a significant impact on the clinical outcomes of OC patients, the subgroup analyses provide an important input. The presented study examined the role of SFAA profiles in differential diagnosis of ovarian tumors and assessed the performance of several multimarker models for pre-surgical evaluation of ovarian masses. A possible direction of future research could be the assessment of SFAA profiles in OC screening, before the actual ovarian tumor is observed in ultrasound examination. Further analyses comparing PFAA alterations in OC and other cancers are also necessary to establish the role of possible new OC biomarkers.

## 5. Conclusions

This is, to the best of our knowledge, the first study analyzing SFAA profiles in differential diagnosis of ovarian tumors. SFAA profiles were proved to be significantly altered in OC cancer patients compared to patients with BOTs. The results of this study indicated that histidine is a possible new OC biomarker. Adding histidine to a multimarker panel together with CA125 and HE4 may improve the differential diagnosis of ovarian tumors. The results of subgroup analyses suggest that citrulline may be indicative of advanced stage and/or type II OC, and cystine may be indicative of early stage and/or type I OC. The findings of this research broaden the knowledge on the metabolic changes of amino acids in OC. Further studies are needed to determine the role of SFAA alterations in OC and select valuable biomarkers for practical use in the future.

## Figures and Tables

**Table 1:** List of analyzed amino acids and biogenic amines in serum.

Full Name	Abbreviation
1-Methyl-L-histidine	1MHis
3-Methyl-L-histidine	3MHis
L- α- Aminoadipic acid	Aad
L-α-Amino-n-butyric acid	Abu
L-Alanine	Ala *
L-Anserine	Ans **
L-Arginine	Arg *
Argininosuccinic acid	Asa **
L-Asparagine	Asn *
L-Aspartic acid	Asp *
D, L-β-Aminoisobutyric acid	bAib
β-Alanine	bAla
L-Carnosine	Car **
L-Citrulline	Cit
Cystathionine	Cth **
L-Cystine	Cys *
Ethanolamine	EtN
γ-Amino-n-butyric acid	GABA **
L-Glutamine	Gln *
L-Glutamic acid	Glu *
Glycine	Gly *
L-Homocitrulline	Hci **
L-Homocystine	Hcy **
L-Histidine	His *
δ-Hydroxylysine	Hyl **
Hydroxy-L-proline	Hyp
L-Isoleucine	Ile *
L-Leucine	Leu *
L-Lysine	Lys *
L-Methionine	Met *
L-Ornithine	Orn
O-Phosphoethanolamine	PEtN
L-Phenylanalanine	Phe *
L-Proline	Pro *
O-Phospho-L-serine	PSer **
Sarcosine	Sar
L-Serine	Ser *
Taurine	Tau
L-Threonine	Thr *
L-Tryptophan	Trp *
L-Tyrosine	Tyr *
L-Valine	Val *

* Twenty basic proteogenic amino acids. ** Nine amino acids excluded from final analysis.

**Table 2:** Study group characteristics.

	Ovarian Cancer			Borderline Tumors	Benign Ovarian Tumours
	Total	Type I	Type II		
Number of samples (%)	38 (24.4)	7 (4.5)	31 (19.9)	6 (3.9)	62 (39.7)
Age (years) median (range)	60 (32–78)	54 (32–70)	63 (36–78)	48 (37–52)	40.5 (17–72)
BMI median (range)	25.1(18.6–38.4)	26.0 (18.6–36.9)	25.0 (20.7–38.4)	27.3 (17.3–31.6)	24.3 (17.9–39.9)
% of postmenopausal	79	57	84	33	26
FIGO stage, *n* (%)					
I	10 (26.3)	4 (57.1)	6 (19.4)	6 (100)	N/A
II	2 (5.3)	0	2 (6.5)	0	N/A
III	25 (65.8)	3 (43.9)	22 (71.0)	0	N/A
IV	1 (2.6)	0	1 (3.2)	0	N/A
Histopathological type, *n* (%)					
Serous	16 (42.1)	3 (7.9)	13 (34.2)	4 (66.7)	14 (22.6)
Endometrioid	4 (10.5)	0	4 (10.5)	1 (16.7)	18 (29.0)
Mucinous	1 (2.6)	1 (2.6)	0	1 (16.7)	2 (3.2)
Clear cell	3 (7.9)	3 (7.9)	0	0	N/A
Undifferentiated	10 (26.3)	0	10 (26.3)	0	N/A
Non identified	4 (10.5)	0	4 (10.5)	0	N/A
Teratoma	N/A	N/A	N/A	N/A	11 (17.7)
Other	N/A	N/A	N/A	N/A	17 (27.4)

**Table 3:** Serum free amino acids and ovarian cancer markers (CA125 and HE4) showing significant *p*-values (*p* < 0.05) and corresponding areas under the receiver operating characteristic (AUC of ROC) curves in differential diagnosis between the analyzed groups. The highest obtained AUC in each group is in bold.

			3.1				3.2				3.3					
		Decreased or Increased in OC	OC (Excl. Borderline Ovarian Tumours) vs. BOT		OC+Borderline Ovarian Tumours vs. BOT		FIGO Stage I-II OC (Incl. Borderline Ovarian Tumours) vs. BOT		FIGO Stage III-IV OC vs. BOT		Type I OC (Incl. Borderline Ovarian Tumours) vs. Type II OC		Type I OC (Incl. Borderline Ovarian Tumours) vs. BOT		Type II OC vs. BOT	
Full Name	Abbreviation		*p*-Value	AUC	*p*-Value	AUC	*p*-Value	AUC	*p*-Value	AUC	*p*-Value	AUC	*p*-Value	AUC	*p*-Value	AUC
L-α-Aminoadipic acid	Aad	D	0.005982	0.663	0.005180	0.659			0.009773	0.674					0.000956	0.709
L-Asparagine	Asn *	D	0.019468	0.640	0.046138	0.614										
L-Citrulline	Cit	D	0.000008 **	0.748	0.000163 **	0.705			0.000001 **	0.807	0.022393 **	0.730			0.000004 **	0.778
L-Cystine	Cys *	I	0.037785	0.624	0.015638	0.638	0.002588 **	0.742					0.038398 **	0.689		
L-Glutamine	Gln *	D	0.001596	0.689	0.006620	0.655			0.003427	0.699					0.005316	0.678
L-Histidine	His *	D	0.000000 **	0.820	0.000000 **	0.787	0.000124 **	0.786	0.000084 ***	0.788			0.000769 **	0.762	0.000001 **	0.798
L-Isoleucine	Ile *	I	0.004064 **	0.638	0.009179 ***	0.620			0.011631 ***	0.670					0.002504 **	0.669
L-Leucine	Leu *	I	0.042357 ***	0.606	0.026877 ***	0.614									0.013807 **	0.646
L-Phenylanalanine	Phe *	I			0.038955	0.618									0.013842	0.657
L-Threonine	Thr *	D	0.011012	0.652	0.021344	0.632			0.011104	0.662						
L-Tryptophan	Trp *	D	0.000103 **	0.718	0.000513 **	0.694			0.000020	0.768					0.000125 **	0.729
cancer antigen 125	CA125	I	0.000000	**0.965**	0.000000	**0.919**	0.000012	**0.840**	0.000000	**0.974**	0.010099	**0.749**	0.000477	**0.810**	0.000000	**0.965**
human epididymis protein 4	HE4	I	0.000000	**0.975**	0.000000	**0.929**	0.000006	**0.853**	0.000000	**0.982**	0.004609	**0.774**	0.000223	**0.828**	0.000000	**0.972**

* Proteinogenic amino acids. ** Based on *t*-test. *** Based on Welch test. The remaining *p*-values are based on Mann–Whitney U test. OC—ovarian cancer, BOT—benign ovarian tumors, D—decreased, I—increased.

**Table 4:** Areas under the receiver operating characteristic (AUC of ROC) curves for multivariate models comparing their diagnostic utility in differential diagnosis between the analyzed groups.

Results Section	Analyzed Groups	2-Marker Model AUC (CI 95%)	3-Marker Model AUC (CI 95%)	
		CA125+HE4	CA125+HE4+Histidine	CA125+HE4+Citrulline
3.1	OC vs. BOT	0.988 (0.965–)	0.995 (0.981)	x
3.1	OC+borderline ovarian tumors vs. BOT	0.938 (0.863–)	0.955 (0.893–)	x
3.2	FIGO stage I-II OC vs. BOT	0.839 (0.682–0.995)	0.873 (0.710–0.987)	x
3.2	FIGO stage III-IV OC vs. BOT	0.996 (0.978–)	x	0.999 (0.996–)
3.3	Type I OC vs. BOT	0.802 (0.523–)	0.822 (0.575–0.998)	x
3.3	Type II OC vs. BOT	0.988 (0.961–)	0.993 (0.974–)	x

OC—ovarian cancer, BOT—benign ovarian tumors.

**Table 5:** Overview of metabolomic studies on ovarian cancer (OC) and their results: concentrations of several amino acids were significantly different in OC compared to benign ovarian tumors (BOTs) and/or healthy controls.

Reference	Metabolite/Sample	Design	Alanine	Aminoadipic Acid	Asparagine	Citrulline	Cystine	Glutamate	Glutamine	Glycine	Histidine	Isoleucine	Leucine	Lysine	Methionine	Phenylalanine	Proline	Serine	Threonine	Tryptophan	Tyrosine	Valine
Zhou et al. 2010 [15]	serum	OC vs. BOT and healthy controls	↑				↑			↑								↑	↑			
Hilvo et al. 2015 [16]	serum	OC (high grade) vs. BOT and healthy controls	↓					↑	↑	↑					↓	↓	↓	↓	↓	↓	↓	↓
Garcia et al. 2011 [17]	serum	OC (early stage FIGO I/II) vs. healthy controls	↓																			↓
Bachmayr-Heyda 2017 [18]	serum	OC (high-grade serous) vs. healthy controls				↓					↓			↓					↓	↓		
Buas et al. 2016 [19]	plasma	OC (serous) vs. BOT (serous)	↓																			
Ke et al. 2014 [20]	plasma	OC vs. BOT/uterine fibromas									↓			↓		↓				↓		
Miyagi et al. 2017 [21]	plasma	OC+borderline tumors vs. BOT									↓	↑					↑			↓		
Zhang et al. 2012 [22]	plasma	OC vs. BOT																		↓		
Our study	serum	OC+borderline tumors vs. BOT		↓	↓	↓	↑		↓		↓	↑	↑			↑			↓	↓		

## Data Availability

Data is contained within the article or supplementary material.

## Supplementary Materials

The following are available online at https://www.mdpi.com/1660-4601/18/4/2167/s1, Table S1: Results of the mass spectrometry analysis of the amino acids and biogenic amines and concentrations of CA125 and HE4. Supplementary Excel sheet with datasets.

## Abbreviations

OC	ovarian cancer
CA125	cancer antigen 125
HE4	human epididymis protein 4
ROC	receiver operating characteristic
AUC	area under curve
BOT	benign ovarian tumor
PFAA	plasma free amino acids
SFAA	serum free amino acids
LOQ	limit of quantitation
BMI	Body Mass Index
FIGO	the International Federation of Gynaecology and Obstetrics
