# Supplementary material for: Serum Free Amino Acid Profiling in Differential Diagnosis of Ovarian Tumors—A Comparative Study with Review of the Literature
Source: Int J Environ Res Public Health. 2021 Feb 23;18(4):2167. doi: 10.3390/ijerph18042167 (PMC7926859; doi:10.3390/ijerph18042167)
Supplement: Supplementary file 1 [file ijerph-18-02167-s001.pdf]

Table S1. Results of the mass spectrometry analysis of the amino acids and biogenic amines and concentrations of CA125 and HE4.

	Amino acid		Benign ovarian tumors (n = 62)		Ovarian cancer (n = 38), borderline tumors (n=6)							
					Type I (incl. borderline tumors)		Type II		Total (excluding borderline tumors)		Total (including borderline tumors)	
	**Full name**	**Abbreviation**	**Mean**	**SD**	**Mean**	**SD**	**Mean**	**SD**	**Mean**	**SD**	**Mean**	**SD**
1	1-Methyl-L-histidine	1MHis	5.62	5.34	11,3692	13,7862	6,6032	5,772	6.70	6.13	8,0114	9,007
2	3-Methyl-L-histidine	3MHis	4.00	1.11	3,9692	1,7806	4,4484	1,956	4.29	1.87	4,3068	1,898
3	L- α- Aminoadipic acid	Aad	0.76	0.25	0,8923	0,5107	1,0355	0,409	1.00	0.45	0,9932	0,440
4	L-α-Amino-n-butyric acid	Abu	28.3	9.71	30,9385	7,1884	30,2065	11,475	30.53	10.73	30,4227	10,315
5	L-Alanine	Ala	416.42	86.01	366,9077	94,3326	411,4065	122,828	396.79	122.32	398,2591	115,891
6	L-Arginine	Arg	103.96	28.64	109,8769	31,2225	105,8742	34,200	106.30	32.80	107,0568	33,037
7	L-Asparagine	Asn	63.18	12.83	56,4154	11,6567	58,0871	14,570	56.64	14.10	57,5932	13,661
8	L-Aspartic acid	Asp	28.80	14.63	30,0231	12,3349	32,9548	20,260	31.59	18.63	32,0886	18,184
9	D, L-β-Aminoisobutyric acid	bAib	2.77	1.05	3,0538	1,3956	3,2161	1,271	3.19	1.35	3,1682	1,295
10	β-Alanine	bAla	28.52	9.67	25,4308	11,2709	41,2516	60,065	38.33	54.76	36,5773	51,047
11	L-Citrulline	Cit	29.02	6.53	27,7077	8,4866	21,6161	7,471	22.22	7.71	23,4159	8,182
12	L-Cystine	Cys	32.37	16.18	42,3846	11,9794	38,4161	19,126	38.96	18.31	39,5886	17,280
13	Ethanolamine	EtN	12.42	6.15	12,4538	6,8220	11,8645	5,045	11.51	4.73	12,0386	5,552
14	L-Glutamine	Gln	674.61	83.27	630,7923	137,1127	615,7387	100,544	607.65	107.89	620,1864	111,120
15	L-Glutamic acid	Glu	82.80	56.86	79,4846	44,9876	98,8161	56,812	93.61	53.95	93,1045	53,817
16	Glycine	Gly	316.85	90.33	300,5231	77,2617	314,2677	96,297	305.12	91.72	310,2068	90,419
17	L-Histidine	His	85.06	11.41	72,4538	13,4150	70,3839	14,645	69.15	14.26	70,9955	14,169
18	Hydroxy-L-proline	Hyp	10.91	4.63	11,2385	9,0932	11,9000	8,073	11.41	7.85	11,7045	8,285
19	L-Isoleucine	Ile	68.92	15.80	74,5846	23,3169	80,7742	20,096	79.74	20.80	78,9455	21,015
20	L-Leucine	Leu	118.79	27.26	128,2077	37,8701	135,6355	36,218	133.33	37.62	133,4409	36,430
21	L-Lysine	Lys	243.06	47.36	229,0462	61,2514	232,6548	49,559	226.01	52.97	231,5886	52,567
22	L-Methionine	Met	24.32	5.14	22,6077	5,5246	23,4935	7,613	23.02	7.42	23,2318	7,009
23	L-Ornithine	Orn	117.00	48.23	107,4462	32,4678	112,5387	48,374	109.36	45.18	111,0341	43,958

24	O-Phosphoethanolamine	PEtN	2.01	1.69	3,1538	3,3979	1,9984	2,257	2.13	2.24	2,3398	2,657
25	L-Phenylanalanine	Phe	69.71	14.23	69,3538	11,1447	78,6065	17,959	76.23	17.54	75,8727	16,671
26	L-Proline	Pro	184.52	57.19	168,0692	73,9707	189,7677	57,468	184.31	63.79	183,3568	62,701
27	Sarcosine	Sar	1.04	1.09	0,7846	0,5984	1,2387	2,583	1.15	2.36	1,1045	2,191
28	L-Serine	Ser	167.75	37.66	163,7923	30,6790	164,2161	50,566	161.86	46.85	164,0909	45,239
29	Taurine	Tau	173.32	56.55	210,2923	84,4489	188,6839	89,855	190.60	85.86	195,0682	87,879
30	L-Threonine	Thr	132.72	34.90	113,9231	28,1896	117,5226	41,112	114.28	38.81	116,4591	37,466
31	L-Tryptophan	Trp	49.20	8.37	45,8308	11,0492	41,0258	10,878	41.31	11.03	42,4455	11,025
32	L-Tyrosine	Tyr	56.07	12.54	55,9462	15,4671	61,0968	22,330	60.01	21.70	59,5750	20,501
33	L-Valine	Val	225.11	45.06	233,6077	60,4087	240,0677	42,173	238.34	49.05	238,1591	47,625
34	cancer antigen 125	CA125	43,2871	67,13660	303,1331	500,2241	964,0339	1275,753	882,0832	1188,266	768,7677	1139,458
35	human epididymis protein 4	HE4	55,1332	15,66240	233,5454	369,7257	567,8990	517,092	530,5703	509,969	469,1127	498,505

SAMPLE No	group	CA125	HE4	PEtN	Tau	Asn
1s	lag	64.2	65.01	1.7	154.6	71.2
2s	rak	320.1	544.0	2.3	194.5	48.5
4s	rak	384.9	776.7	0.8	182.1	53.5
5s	lag	9.1	43.61	1.7	141.9	53.4
6s	lag	7.7	32.81	1.4	111.9	51.7
7s	lag	45.47	43.85	2.4	148.8	56.3
9s	lag	25.13	44.68	1.2	66.0	58.2
12s	lag	71.23	45.84	0.1	125.7	81.2
15s	lag	28.28	42.47	1.9	143.0	59.6
16s	rak	2608.0	844.7	3.8	177.2	84.6
17s	lag	53.85	65.01	3.1	187.7	66.0
23s	rak	241.6	500.3	1.4	167.2	33.3
24s	lag	14.25	103.7	2.2	214.1	49.7
25s	lag	204.5	45.07	3.2	176.5	63.1
26s	rak	71.5	67.14	1.4	223.1	66.0
27s	lag	43.46	37.44	1.2	160.8	57.1
29s	lag	10.97	73.2	4.6	231.4	95.6
31s	lag	91.75	50.59	0.9	131.4	57.4
33s	lag	53.85	65.01	1.9	110.6	73.5
34s	lag	14.58	74.29	2.1	214.9	50.3
35s	lag	34.95	53.08	0.4	142.4	60.8
39s	lag	19.44	41.49	1.1	88.1	41.5
40s	lag	29.9	42.35	1.6	84.0	53.2
41s	lag	12.1	40.7	0.8	92.0	49.8
42s	lag	42.93	38.16	1.5	91.3	63.5
43s	lag	30.49	53.5	1.3	68.0	35.4
44s	lag	10.01	43.66	1.4	134.3	66.1
48s	lag	6.12	56.88	1.3	164.0	62.9
50s	lag	44.07	31.95	1.8	178.9	53.3
51s	lag	8.78	51.3	10.0	98.6	52.5
52s	lag	17.78	37.2	1.4	137.8	48.9
53s	lag	11.71	42.75	2.0	105.6	60.3
55s	lag	11.1	44.23	3.7	199.0	61.6
58s	rak	5000.0	1500.0	1.6	55.5	37.4
62s	lag	14.52	61.84	1.1	155.8	49.9
64s	lag	11.65	44.46	4.2	225.3	73.4
65s	lag	8.92	50.16	0.8	218.7	75.5
66s	rak	1837.0	1386.0	0.8	182.1	47.5
67s	lag	57.0	65.01	2.9	228.0	70.6
68s	lag	17.09	56.02	1.4	306.5	80.7
71s	lag	20.95	77.75	0.25	158.4	103.0
72s	lag	50.14	120.8	0.25	183.5	75.8
73s	rak	318.9	106.6	1.3	78.2	39.7
74s	rak	465.2	55.35	2.3	290.0	62.8
78s	lag	24.19	58.08	3.3	171.9	60.8
80s	rak	671.1	300.9	0.9	148.4	65.7
81s	rak	78.47	102.4	1.3	115.3	41.5
83s	rak	642.4	679.7	3.1	178.9	48.9
85s	lag	48.22	55.97	2.8	139.6	55.0

86s	rak	637.7	172.6	0.5	54.6	66.7
88s	rak	1328.0	148.1	2.3	166.4	50.3
93s	rak	511.2	1500.0	2.5	109.4	60.1
94s	lag	12.72	74.88	1.4	144.3	51.1
95s	rak	762.8	665.3	0.9	181.9	42.5
96s	rak	112.4	1500.0	1.8	281.6	62.2
97s	lag	13.2	65.01	1.2	165.8	54.3
98s	lag	156.7	74.7	6.5	337.9	74.8
99s	lag	7.11	54.84	2.3	185.8	53.4
101s	rak	63.21	120.4	1.9	156.4	52.6
102s	lag	28.64	42.38	1.6	140.3	51.7
104s	lag	53.85	65.01	3.8	225.5	61.9
106s	lag	61.17	55.91	2.4	166.7	70.9
107s	lag	63.11	62.99	2.5	195.8	46.4
108s	rak	54.23	339.1	0.7	244.4	70.4
109s	lag	53.85	65.01	3.0	151.8	52.9
110s	rak	789.7	303.0	1.9	230.5	62.3
114s	lag	3.75	53.07	2.5	273.4	78.7
115s	rak	527.6	183.2	0.25	44.4	45.2
116s	rak	683.8	228.7	9.9	321.3	50.3
117s	rak	3771.0	1500.0	0.6	60.3	64.7
121s	lag	7.38	62.27	4.2	215.5	72.4
122s	rak	80.2	97.5	5.6	233.2	67.4
123s	rak	141.2	99.37	2.3	134.1	43.0
126s	rak	768.0	205.7	1.6	81.5	64.2
128s	rak	211.9	509.0	8.8	326.7	91.3
129s	rak	693.5	116.6	1.0	257.7	38.0
131s	lag	102.7	49.86	2.1	256.4	73.9
132s	rak	181.4	140.8	2.5	162.9	50.5
133s	lag	53.85	54.89	2.1	200.0	55.0
134s	rak	321.2	185.7	6.2	291.6	57.8
135s	rak	46.7	838.3	0.25	215.8	48.0
137s	rak	793.5	381.1	0.2	328.4	87.2
138s	rak	290.7	106.1	0.4	180.8	66.5
139s	lag	36.18	47.79	0.25	108.0	82.7
140s	rak	2009.0	1500.0	2.0	252.4	51.0
142s	lag	53.47	44.56	5.6	244.3	74.7
150s	lag	24.3	68.66	1.7	231.1	62.6
151s	lag	23.87	49.14	0.1	186.9	77.4
152s	lag	53.85	65.01	1.1	241.2	63.8
153s	rak	282.2	414.7	0.25	252.2	62.1
154s	lag	486.9	68.2	0.25	223.9	72.9
163s	lag	17.01	44.6	0.1	213.1	84.7
168s	lag	53.85	65.01	1.1	192.2	61.7
169s	lag	8.75	34.68	0.6	221.1	75.5
170s	rak	4257.0	1500.0	4.8	397.5	74.9
172s	rak	1508.0	477.6	0.4	114.0	36.5
173s	lag	10.53	47.82	0.2	140.8	53.5
175s	rak	53.85	65.01	0.5	170.4	57.1
176s	lag	15.74	44.67	0.3	169.2	57.9

178s	lag	10.94	57.38	2.8	223.8	53.7
49s	bor	33.24	61.26	1.5	132.6	54.2
56s	bor	33.24	146.4	2.0	237.2	61.8
70s	bor	26.3	28.8	1.2	146.8	66.3
79s	bor	116.00	128.2	2.5	177.1	68.9
105s	bor	11.22	61.26	1.7	229.9	53.4
127s	bor	86.62	53.37	13.0	416.5	77.3

Ser	Gly	Hyp	EtN	Gln	Asp	Cit	Thr	Sar
182.2	264.1	12.2	7.9	725.5	23.6	27.9	154.7	1.6
153.7	194.2	10.9	9.6	668.9	20.6	32.6	127.1	0.7
132.9	238.4	47.4	8.8	565.4	42.6	25.6	110.5	0.9
122.4	237.1	9.4	6.8	652.1	21.9	28.4	109.1	0.4
133.0	330.5	10.8	6.9	652.1	10.7	23.9	130.8	0.4
170.4	314.4	9.0	7.9	634.3	17.7	31.4	134.1	0.4
145.6	281.4	18.9	12.1	721.1	14.6	29.0	154.5	0.5
196.3	298.4	16.8	9.2	692.8	22.9	35.6	164.5	1.1
180.7	396.9	6.8	7.5	795.3	14.2	28.0	136.7	0.5
297.3	528.3	18.1	13.3	725.1	59.8	30.0	246.5	1.5
178.4	261.0	14.5	10.1	561.6	36.7	23.0	117.0	0.8
76.7	126.1	10.5	5.5	535.1	15.4	15.7	86.5	0.3
185.3	230.5	14.6	18.5	838.9	28.3	41.6	71.1	1.3
185.4	261.4	13.1	35.6	681.1	21.1	30.9	146.2	0.7
150.0	240.0	9.0	9.2	547.9	63.9	17.2	164.5	0.5
149.9	342.6	9.3	9.1	680.3	22.5	22.0	153.5	0.7
251.6	554.4	26.7	15.2	767.2	50.2	26.0	193.0	1.5
139.5	283.0	9.1	8.7	648.1	26.4	34.9	201.9	0.7
234.9	346.3	9.8	6.0	764.2	7.5	21.1	151.8	5.1
151.1	286.5	7.1	10.9	751.4	24.6	33.5	105.9	0.8
160.6	357.5	5.0	10.4	795.5	24.3	27.1	133.3	0.1
89.4	144.7	4.6	12.1	368.2	17.6	14.9	79.0	0.1
135.1	265.5	6.5	13.9	640.7	27.1	23.2	145.0	0.4
127.2	244.9	9.7	7.1	771.5	15.8	26.1	117.1	0.8
175.6	293.7	5.9	11.8	733.7	20.3	25.3	138.5	0.7
92.4	218.4	6.8	7.6	713.1	5.4	28.2	70.0	1.3
135.8	251.4	11.9	7.6	631.6	20.3	30.9	120.4	1.3
164.4	512.7	9.5	8.4	644.4	23.1	28.1	119.4	0.6
141.1	214.8	11.4	8.9	551.8	24.3	19.0	100.2	1.0
130.5	319.0	7.8	8.5	739.5	13.6	33.5	94.3	1.4
124.3	278.9	10.0	6.4	660.1	19.9	26.6	104.3	0.5
172.3	313.4	7.5	7.9	727.5	7.1	19.4	161.9	0.9
173.5	350.0	11.0	10.4	712.5	24.8	33.1	203.5	0.8
92.6	388.2	16.7	4.3	665.5	5.6	10.3	67.4	1.2
90.0	210.8	21.0	9.4	575.8	41.6	27.6	107.0	2.7
189.5	328.0	8.9	17.1	647.1	48.4	26.5	108.6	0.4
205.9	371.3	10.6	16.1	605.3	62.6	24.4	110.2	1.4
147.1	283.8	5.3	12.7	655.9	38.1	24.9	85.0	0.3
166.3	283.4	9.0	14.7	770.7	36.0	27.3	118.7	2.0
256.1	636.9	15.2	20.6	774.9	55.5	35.0	175.6	1.4
180.8	383.7	13.7	14.6	686.9	55.3	24.8	100.0	0.7
215.1	461.5	8.6	15.0	598.6	63.6	29.9	115.5	0.7
119.3	241.8	10.8	7.8	621.3	21.7	24.8	85.7	0.4
243.2	255.8	4.6	15.1	458.9	75.3	11.9	97.7	0.3
192.1	436.6	12.4	8.0	671.8	16.4	30.5	154.7	1.3
168.0	275.9	9.7	10.3	654.4	36.9	27.6	121.5	1.2
126.5	259.6	8.3	7.8	575.9	31.3	31.5	71.5	0.7
145.2	238.2	18.3	6.8	541.5	17.4	14.4	94.2	0.7
95.1	189.7	3.9	8.3	529.2	8.8	27.3	75.6	0.1

121.6	207.3	14.9	17.6	710.7	7.6	20.3	137.3	15.0
166.3	282.3	10.5	8.5	584.5	27.6	24.9	105.9	1.1
114.8	208.0	17.7	9.6	757.6	9.8	8.8	54.4	1.0
141.8	265.1	6.3	8.3	618.9	17.1	26.5	96.1	1.2
106.4	171.7	14.0	9.8	511.0	33.9	23.0	74.1	1.4
147.3	345.6	16.2	9.7	730.8	25.9	25.3	162.7	1.3
161.5	405.6	10.8	14.9	770.1	24.9	40.1	114.9	0.7
187.4	362.9	9.7	11.5	730.6	22.9	22.7	144.4	0.5
137.5	207.0	8.9	7.1	731.5	21.5	29.0	92.9	1.7
146.3	239.3	22.6	5.4	716.7	25.6	32.1	124.2	1.2
156.0	189.8	8.2	9.7	491.9	26.3	20.1	82.2	0.9
177.6	413.1	23.7	9.4	769.8	27.7	36.3	146.1	1.5
204.1	320.0	9.9	10.6	723.6	30.0	26.8	162.2	0.6
141.9	232.1	10.7	9.4	622.0	28.5	34.5	112.5	0.7
216.1	400.2	12.2	16.8	624.3	56.0	22.2	122.4	0.8
135.6	258.2	10.1	8.1	625.2	16.9	23.7	111.3	1.1
203.1	235.8	4.6	12.8	577.8	35.6	19.5	147.4	0.3
193.8	323.6	10.3	18.1	687.5	51.2	46.3	187.4	1.3
132.2	233.8	9.8	6.5	452.3	34.3	13.0	92.8	1.0
169.2	273.4	9.0	13.6	568.5	37.3	16.1	96.9	0.2
149.1	415.2	18.1	7.2	690.7	19.7	19.5	152.6	1.1
205.7	404.5	23.5	9.9	528.2	44.2	33.9	219.3	0.6
173.1	374.1	9.2	10.1	579.7	26.1	33.1	117.5	2.3
134.1	357.6	4.8	8.9	548.8	10.8	15.2	80.7	0.7
189.6	336.8	8.6	24.2	768.5	12.4	27.9	147.1	0.9
214.3	314.1	9.5	14.6	708.4	51.4	31.3	177.2	0.5
129.6	244.7	2.0	12.3	342.2	20.8	10.0	66.0	0.1
185.6	338.4	7.3	14.5	643.2	51.1	24.3	140.6	1.2
164.8	451.0	5.1	8.1	826.4	11.3	24.0	121.4	1.0
158.5	259.4	6.6	11.5	570.5	39.7	17.7	134.6	0.4
190.7	273.8	3.8	11.5	418.0	25.8	16.9	94.0	0.1
149.6	295.2	5.6	14.7	449.2	32.4	18.9	89.3	0.4
280.1	484.7	9.2	23.3	605.1	80.4	27.1	149.6	0.5
154.6	297.4	7.1	15.1	562.6	36.2	19.4	117.0	0.6
183.6	238.2	6.8	16.0	650.0	26.6	24.2	127.7	7.4
176.9	334.2	9.7	15.3	569.8	30.2	20.3	95.7	0.5
207.4	300.8	13.1	12.0	653.0	38.1	31.1	131.9	1.1
139.6	284.8	6.6	14.7	623.7	13.1	25.1	148.1	0.2
186.9	337.7	11.4	13.3	707.0	39.2	42.0	168.8	0.7
135.4	257.0	9.6	34.2	605.1	37.0	26.3	100.5	0.5
172.1	289.8	7.6	15.1	716.8	29.5	32.5	128.2	0.7
228.2	369.4	13.1	28.3	693.7	41.4	32.7	174.3	0.5
215.0	452.7	12.7	16.1	682.7	56.4	43.3	219.0	0.4
210.5	382.7	11.0	20.5	716.7	26.1	35.3	117.6	0.4
216.1	394.3	8.8	25.5	725.7	56.2	26.8	162.2	1.1
185.9	461.1	19.7	19.8	664.9	56.7	17.1	163.6	0.5
105.3	365.3	4.4	6.3	484.0	3.5	17.1	61.4	0.1
160.8	384.0	12.1	9.4	768.4	22.2	35.0	117.8	0.8
204.9	431.3	8.0	9.2	705.5	30.9	42.5	105.2	1.5
185.9	264.8	7.0	10.6	683.1	36.2	26.3	125.3	0.7

124.5	243.4	19.0	9.1	687.0	16.2	43.0	113.4	1.2
166.8	285.6	10.7	8.6	674.4	26.2	24.4	111.1	0.7
173.7	232.6	36.0	11.2	840.8	32.8	28.5	167.2	0.9
156.1	467.8	14.3	10.3	796.3	23.7	30.0	145.7	0.5
167.2	325.8	6.4	10.5	714.7	26.5	32.0	137.5	1.4
161.5	375.7	7.9	32.4	599.2	35.5	25.7	94.6	0.9
244.2	367.6	6.2	19.5	572.2	66.9	45.2	125.4	0.7

bAla	Ala	Glu	His	1MHis	3MHis	Aad	bAib	Abu
35.8	422.9	36.1	94.8	2.0	3.7	0.8	2.4	28.7
30.0	511.4	54.3	76.0	4.4	5.5	0.8	3.2	28.6
60.9	376.4	76.7	48.2	14.1	5.6	1.2	3.1	13.8
41.9	324.9	37.0	96.7	0.9	3.8	0.3	2.4	20.3
13.2	481.5	64.2	74.2	2.8	5.3	0.9	3.1	44.7
33.9	342.4	41.7	82.4	7.9	3.7	0.7	3.2	40.9
33.5	442.0	26.7	76.5	2.6	3.8	0.9	2.8	38.5
29.9	505.9	49.6	85.6	1.8	4.3	0.7	1.5	39.9
26.7	393.5	37.2	88.8	3.7	3.7	0.8	1.9	31.0
32.6	573.5	111.9	82.0	4.6	4.7	0.8	4.5	43.8
35.9	371.9	72.3	80.7	17.0	5.6	0.9	2.9	32.5
27.8	364.7	84.1	63.9	5.5	12.6	1.6	5.0	19.7
30.8	401.1	96.6	63.1	1.7	2.5	0.6	2.3	7.9
24.3	367.9	39.6	86.7	14.1	3.7	0.6	2.2	31.9
33.1	336.2	94.3	73.3	3.3	2.7	0.6	1.3	10.8
28.6	391.4	37.2	84.6	18.6	4.0	0.9	4.0	43.3
36.3	565.2	142.8	111.8	11.3	5.2	1.0	2.8	27.6
22.5	354.9	65.0	83.4	6.8	3.9	0.9	5.5	23.9
29.8	505.7	20.9	98.4	5.0	3.4	0.5	2.5	22.9
44.5	446.1	71.6	86.0	2.9	6.4	0.8	3.2	22.1
4.9	444.6	55.3	95.9	3.1	3.9	0.4	3.5	32.0
30.2	227.5	35.3	50.8	1.9	2.1	0.6	1.5	16.2
32.7	388.7	35.7	81.5	1.0	2.9	0.6	2.5	22.4
35.6	379.1	22.5	77.0	3.2	2.9	0.5	2.4	27.1
27.5	371.6	52.3	91.3	1.4	3.5	1.1	3.8	66.6
24.9	293.2	9.2	65.2	1.8	3.5	0.4	1.8	13.0
26.1	270.9	37.8	86.5	1.2	4.8	0.6	3.3	24.3
28.3	410.5	77.8	81.1	8.8	5.2	1.4	3.6	34.2
30.7	268.6	42.1	84.1	2.5	3.9	0.5	2.9	27.5
51.2	269.6	27.4	80.4	17.0	5.2	0.8	2.3	27.6
27.3	373.0	45.2	70.1	1.5	3.5	0.6	2.0	23.7
39.4	426.8	26.0	90.6	21.6	4.1	1.3	3.9	39.9
30.0	411.4	69.8	78.3	2.6	4.8	0.9	1.8	19.6
40.7	546.5	85.8	47.9	2.1	3.5	0.8	2.4	23.1
20.4	325.5	174.0	71.4	4.0	3.0	0.4	3.5	11.8
24.7	411.6	145.3	108.1	2.5	2.9	0.6	2.2	22.5
39.3	456.8	193.5	91.8	1.4	4.5	0.7	3.1	39.2
32.0	340.3	126.2	65.2	2.5	3.1	0.7	2.9	30.1
37.1	429.9	102.9	96.9	0.9	4.4	0.5	4.1	26.2
25.2	545.2	181.1	101.3	5.4	4.9	0.6	2.9	24.9
26.2	525.8	214.0	91.2	0.7	3.3	0.6	1.4	18.0
11.5	441.6	209.7	92.4	3.2	4.6	0.9	6.6	34.7
37.9	341.8	38.1	60.5	8.7	3.9	0.6	3.0	26.7
39.6	456.2	117.3	51.8	0.1	2.9	1.5	4.0	56.4
40.4	450.0	48.8	76.0	16.0	3.6	0.9	2.8	24.7
41.9	400.9	74.3	72.9	10.3	7.0	1.0	3.2	32.8
15.0	352.9	132.7	69.7	21.7	5.6	1.9	1.7	20.7
29.9	480.4	74.7	67.8	17.6	4.3	0.9	1.4	27.5
4.8	301.6	26.2	67.3	1.3	3.9	0.5	1.2	21.5

359.2	313.3	22.5	72.7	3.2	3.3	0.5	2.8	28.7
28.7	329.1	55.4	68.3	2.9	4.2	1.0	2.8	37.4
35.0	709.3	76.1	79.7	6.5	4.7	1.5	3.0	19.1
21.2	452.1	71.9	84.1	11.5	8.2	1.3	3.8	32.0
35.6	361.0	109.0	47.4	12.7	4.6	2.0	5.7	16.3
27.0	343.9	86.6	59.5	2.2	6.2	0.9	5.6	26.9
27.6	368.2	45.0	80.5	9.6	3.8	0.6	4.0	22.8
8.9	607.4	56.3	101.4	1.8	3.9	0.5	1.1	13.8
31.3	456.9	47.8	73.8	5.9	4.9	0.6	1.8	31.2
37.5	290.9	40.5	54.6	4.7	2.0	0.9	1.6	27.4
28.5	349.8	44.6	69.1	0.5	2.5	0.6	2.0	17.7
17.9	458.7	68.1	85.9	5.9	4.9	0.8	2.9	22.5
26.5	349.2	67.7	78.7	6.7	2.5	0.5	1.6	27.6
24.4	350.3	74.2	84.9	6.2	2.7	0.4	1.5	21.0
44.2	550.9	182.7	90.6	16.3	4.6	0.9	3.4	40.8
34.1	440.0	47.4	99.7	3.4	5.0	0.8	1.7	32.6
32.0	519.0	100.7	70.1	5.0	3.2	1.9	5.0	32.7
28.9	590.5	233.4	97.9	16.6	3.7	1.1	2.8	30.7
37.9	250.5	39.7	53.3	2.1	2.7	0.9	3.6	27.4
23.1	236.2	161.7	57.1	3.8	3.2	1.2	3.2	31.0
34.5	555.2	31.3	83.1	6.4	3.9	1.0	1.3	21.2
27.5	470.2	118.7	87.7	2.6	5.5	0.7	2.4	33.2
30.9	392.5	88.9	75.8	3.3	5.2	2.2	6.8	46.7
22.9	236.6	24.1	64.8	0.8	3.1	1.1	2.7	37.7
23.8	435.4	25.4	91.0	11.1	4.6	0.7	2.7	43.0
32.0	513.7	155.1	109.0	2.8	3.7	0.8	4.8	42.1
3.8	179.5	54.9	43.6	1.6	2.4	0.4	2.1	30.0
39.6	365.9	149.6	85.9	4.8	3.1	0.9	3.7	31.2
18.4	375.1	45.6	69.0	1.8	4.0	0.7	2.8	25.9
30.3	382.0	111.2	72.6	2.2	4.3	0.8	5.4	49.7
5.5	268.1	90.8	70.1	24.7	3.0	0.4	1.9	34.1
19.4	331.2	150.1	71.5	3.3	2.4	1.3	3.9	38.6
47.3	648.1	262.8	77.5	1.9	3.3	0.8	1.6	26.0
21.1	422.0	161.3	93.5	3.9	4.9	0.8	3.5	50.2
32.7	479.7	62.0	89.8	2.5	3.8	1.1	2.5	31.2
12.3	275.3	90.0	67.5	4.5	5.9	1.2	5.0	50.7
41.8	369.5	138.2	73.2	1.8	3.2	0.8	2.9	42.8
4.9	338.6	86.7	78.2	2.1	2.7	0.4	2.8	19.6
21.6	583.8	101.8	93.7	3.8	3.7	0.7	1.8	25.2
26.8	486.0	136.1	88.2	2.6	4.0	0.8	2.3	27.4
34.7	451.6	135.3	81.7	4.0	2.9	0.6	1.8	19.3
29.2	458.9	125.7	89.9	4.0	3.7	0.8	2.7	30.9
33.5	502.2	198.0	104.0	7.5	4.6	1.3	3.8	23.2
34.9	403.3	98.2	71.4	2.8	2.2	0.6	2.7	19.5
43.7	649.4	199.8	97.3	5.4	2.5	1.0	1.7	31.7
36.2	421.0	190.6	70.5	3.9	4.4	0.8	2.4	17.8
2.8	207.8	29.4	50.3	7.7	2.5	0.6	2.0	28.8
10.0	375.6	54.6	89.4	5.0	3.6	1.0	1.6	25.8
29.2	379.7	76.1	76.3	18.6	6.7	0.6	3.5	26.2
28.1	428.1	71.2	89.1	15.3	4.3	1.1	3.5	31.7

28.3	361.0	54.9	84.1	15.6	6.6	0.9	3.1	25.9
22.2	355.3	64.1	86.8	4.0	3.1	1.0	2.2	37.0
34.9	505.0	91.1	84.7	50.6	8.9	1.7	2.5	38.6
13.5	430.2	51.1	87.2	11.7	4.2	0.6	3.6	19.8
25.0	307.2	50.6	85.8	8.0	3.6	0.8	2.4	29.4
29.0	424.8	79.1	73.6	20.7	3.6	0.8	2.7	21.0
28.4	422.8	203.6	78.0	2.9	3.1	0.7	4.8	32.8

Arg	Pro	Orn	Cys	Lys	Met	Val	Tyr	Ile
134.0	223.2	110.4	42.0	247.8	25.5	245.0	67.0	76.6
82.3	198.3	122.8	56.6	281.9	24.8	303.8	66.2	86.1
150.6	191.5	120.4	50.7	203.2	18.2	211.4	61.0	85.1
109.2	104.2	70.3	37.3	259.1	18.0	180.5	42.4	49.5
87.8	183.5	88.2	48.8	257.8	23.7	229.2	64.6	74.4
101.9	202.8	82.8	43.6	241.7	24.4	229.1	54.2	67.3
122.5	206.8	68.8	30.1	217.1	26.1	255.6	60.0	79.2
130.1	171.4	71.6	24.7	245.7	31.2	244.2	60.8	70.6
110.8	212.2	85.1	33.1	219.2	26.5	231.0	66.6	73.0
133.3	274.8	145.5	54.9	271.3	35.9	233.8	77.3	69.0
87.0	179.6	87.4	34.3	198.7	24.3	252.9	42.7	81.2
78.6	176.2	94.8	51.5	203.5	13.8	235.6	59.1	69.6
132.4	357.3	187.8	45.1	173.2	18.5	122.9	57.7	48.8
117.7	179.2	97.5	27.5	298.2	29.1	247.2	46.5	81.1
123.9	116.3	61.3	36.7	257.5	21.7	222.7	44.4	63.6
99.7	152.1	81.4	24.2	249.6	24.8	222.0	45.6	57.2
100.3	349.5	223.7	24.2	327.2	43.1	286.7	75.1	103.3
95.3	192.5	124.4	30.3	255.7	20.2	263.0	54.0	73.3
104.8	330.9	71.8	41.3	282.7	34.7	211.9	64.0	87.6
98.7	139.0	135.7	75.1	297.0	21.7	255.7	68.5	68.4
117.7	126.6	74.8	50.8	216.9	23.2	213.5	55.5	50.8
69.0	89.4	39.7	25.1	140.4	14.2	133.2	27.1	41.5
120.2	124.1	55.9	41.8	174.5	18.5	196.1	37.9	55.6
98.5	153.3	73.8	29.9	165.3	18.6	177.3	43.1	49.6
132.3	171.5	71.8	56.5	299.4	29.5	318.4	64.8	108.1
71.9	150.4	49.3	33.8	147.6	14.9	148.7	41.9	42.8
102.0	182.1	70.8	33.7	187.9	23.5	238.6	33.7	70.5
105.4	183.9	86.4	58.8	278.4	27.4	261.5	56.6	83.7
73.9	143.6	83.9	38.8	214.3	21.8	226.0	50.6	72.6
120.4	108.1	79.0	53.9	216.8	18.4	174.5	47.6	41.5
114.5	106.1	85.8	34.5	182.1	18.4	148.0	49.2	43.2
124.0	187.0	86.8	52.5	247.6	25.0	263.8	64.5	90.1
132.0	202.2	100.0	34.1	232.4	25.9	239.6	54.0	74.5
93.6	243.0	65.6	58.6	173.9	13.9	203.7	36.4	70.5
97.0	300.6	90.9	34.6	181.4	20.6	191.7	49.5	54.7
56.1	170.0	165.1	35.2	253.1	27.4	227.2	49.3	71.1
87.6	146.7	195.1	33.9	319.9	25.0	280.6	60.5	87.0
64.2	219.4	115.7	34.4	175.6	18.4	210.2	64.8	68.2
80.4	152.7	162.1	20.2	244.8	23.6	220.4	54.1	66.3
92.9	180.2	195.2	38.3	285.0	29.6	258.1	62.4	72.6
131.1	222.9	125.9	19.3	229.4	22.9	220.6	57.2	65.6
106.8	207.8	170.8	27.8	260.5	29.6	287.7	61.7	88.2
139.2	140.3	57.4	46.4	149.8	14.6	163.5	40.9	60.8
103.6	147.4	177.2	45.5	270.2	29.0	334.4	65.4	122.7
119.2	199.6	106.1	36.3	277.0	24.9	219.8	59.0	60.2
129.2	167.7	81.3	54.8	246.7	25.7	264.2	61.1	91.2
90.9	183.5	114.2	52.0	245.8	15.3	246.8	66.4	67.4
82.4	206.9	108.3	40.6	220.3	21.6	224.8	59.7	67.4
69.2	88.7	84.4	22.7	180.1	15.9	160.1	35.3	46.7

73.6	201.4	80.2	52.3	178.2	49.7	135.9	159.8	37.5
102.7	192.4	90.9	56.1	205.5	21.8	249.4	59.1	76.1
71.4	343.1	56.1	45.1	216.0	22.0	225.0	63.7	77.9
98.0	192.7	121.2	35.9	282.5	22.2	247.3	58.1	72.5
105.2	231.2	101.8	40.2	174.7	12.9	224.6	85.4	54.8
154.9	199.7	124.6	68.6	277.6	24.1	226.4	61.4	101.5
127.0	164.9	117.4	46.0	243.4	21.0	112.2	54.2	57.5
132.5	251.5	120.2	25.7	241.8	27.3	228.9	72.4	61.2
97.2	246.2	94.9	29.6	184.3	23.7	215.5	61.6	64.3
139.7	121.0	101.1	48.5	235.4	19.1	215.6	54.2	64.4
70.0	121.1	60.6	22.5	156.1	16.3	162.8	41.9	41.7
118.2	161.9	151.6	52.1	290.5	25.7	258.8	62.3	74.6
78.3	152.7	86.8	31.2	235.8	27.0	197.3	53.1	59.4
89.0	161.5	110.1	43.5	208.0	18.0	165.8	42.5	42.1
70.2	249.1	155.5	46.6	296.0	27.5	266.7	69.5	79.0
62.8	185.1	135.2	51.4	245.4	22.4	263.8	64.4	74.3
86.7	154.2	127.3	28.3	285.1	28.0	280.4	70.1	82.0
87.0	237.4	258.3	16.2	311.6	33.7	315.8	97.4	94.9
148.8	163.9	86.3	18.1	189.3	21.6	299.2	57.1	111.4
101.2	137.5	78.6	20.4	167.2	17.8	250.9	39.1	118.2
156.4	205.9	78.5	31.2	317.2	31.9	248.1	83.1	90.6
69.5	199.2	199.0	58.4	283.9	29.3	237.8	56.9	66.6
114.1	308.0	89.0	58.6	250.9	33.6	364.9	88.3	127.5
70.3	100.2	46.9	42.0	171.2	17.5	240.9	44.7	77.9
140.9	144.8	65.2	38.2	253.9	28.7	235.6	56.4	65.2
139.2	238.9	200.5	61.7	360.8	33.2	270.2	56.5	83.7
102.1	53.0	69.2	20.5	108.1	15.2	154.3	33.3	61.1
98.5	133.3	133.8	18.0	232.5	26.8	229.7	54.5	72.8
101.6	168.4	86.9	60.2	247.0	18.7	242.5	50.9	70.0
92.9	178.0	90.5	31.3	261.2	21.2	251.3	58.3	91.1
115.7	81.1	111.8	24.4	174.3	20.8	202.5	38.7	58.1
61.9	140.4	129.6	2.8	201.3	19.5	284.0	46.7	107.4
78.5	304.9	268.2	3.4	254.8	27.3	240.9	72.0	83.8
52.5	149.2	149.4	13.9	261.7	27.5	313.8	59.2	100.1
140.1	307.4	78.6	3.5	261.2	31.7	265.3	90.9	90.0
112.7	194.8	103.0	22.4	237.9	20.6	262.0	44.4	102.9
130.7	138.7	132.2	32.6	249.3	23.6	223.6	45.8	78.0
112.1	130.6	111.9	0.5	189.2	24.3	168.8	52.7	50.4
105.0	211.5	158.6	1.5	262.2	24.1	224.3	51.4	66.3
103.7	218.0	131.5	1.1	275.2	22.4	274.0	51.5	79.2
92.0	193.0	161.3	2.6	193.6	22.7	187.2	51.9	54.4
226.0	163.6	152.1	2.0	322.2	30.1	231.5	61.7	82.9
88.6	208.6	206.1	1.3	268.5	26.3	211.5	62.6	71.8
62.1	176.9	158.6	6.5	194.9	22.7	157.8	33.9	57.8
47.1	234.7	228.3	13.1	316.4	31.0	285.6	78.8	89.6
78.9	221.5	166.5	13.5	227.9	24.7	199.5	56.5	93.0
96.3	65.7	64.6	19.6	136.9	15.4	138.8	32.2	49.8
76.5	175.6	140.7	12.7	238.8	19.2	244.9	65.5	69.6
200.1	175.3	98.2	58.4	266.1	20.1	242.7	43.5	80.3
174.2	161.6	130.3	46.7	340.0	22.3	256.3	63.8	68.7

104.3	145.8	104.8	49.6	269.5	24.6	244.0	54.8	67.2
56.0	150.1	134.1	47.7	244.8	20.9	199.7	55.4	54.3
89.8	283.9	151.7	47.0	316.1	28.5	297.3	76.7	99.5
138.4	164.1	79.2	48.1	248.5	24.2	194.5	44.6	58.6
135.6	143.1	100.7	34.2	297.0	24.3	222.5	49.8	64.4
96.4	115.1	91.7	32.6	220.8	20.5	233.0	57.0	58.2
154.9	207.5	172.4	52.0	274.4	29.0	275.1	57.4	108.4

Leu	Phe	Trp
126.2	66.1	52.1
158.4	74.3	54.4
115.7	88.5	44.4
91.9	57.9	51.2
115.4	62.0	51.9
127.3	60.1	49.8
129.6	63.0	53.5
113.1	67.6	58.0
116.2	60.6	53.0
138.1	100.2	44.0
127.9	68.0	53.4
110.4	63.4	43.7
61.2	50.4	22.6
123.0	63.5	60.0
129.2	86.2	54.4
112.8	62.0	41.3
161.2	88.9	69.2
126.4	59.7	43.4
126.5	72.7	45.8
126.3	72.9	53.4
108.7	73.4	55.2
68.7	42.0	41.1
105.4	58.0	41.1
83.7	54.4	32.8
185.3	70.8	51.4
68.8	52.6	44.4
124.4	62.9	47.9
150.9	72.0	48.1
123.6	67.8	46.9
80.9	49.9	49.4
76.0	54.5	37.3
129.5	73.0	53.4
116.9	56.8	47.8
81.6	47.7	30.5
100.5	70.8	51.8
129.7	77.8	56.1
174.1	115.3	53.3
109.0	73.4	33.6
128.9	85.4	57.4
144.2	88.0	55.2
125.6	94.0	51.9
175.1	110.5	50.2
85.1	51.1	32.0
218.9	112.4	32.9
98.9	62.4	52.3
153.6	78.1	51.6
116.7	67.7	56.7
102.9	64.9	40.5
66.2	50.6	51.9

56.2	93.0	60.7
133.4	66.9	53.2
112.9	58.0	33.6
112.2	70.2	58.9
100.8	90.5	41.2
142.9	67.3	44.1
115.4	64.0	48.0
94.7	74.3	43.9
101.9	70.8	37.0
98.8	57.1	47.5
83.1	59.8	42.0
130.6	72.3	52.9
104.0	86.0	47.6
86.2	58.7	40.6
155.2	98.4	41.1
122.8	67.1	56.1
185.4	89.5	44.3
178.8	98.1	62.8
170.2	85.8	34.9
178.5	70.1	30.1
146.3	79.2	55.4
123.3	71.8	52.7
210.1	82.3	61.1
139.8	55.8	27.4
123.3	59.2	45.7
168.4	92.8	48.3
96.8	62.1	27.2
129.6	75.3	46.5
116.5	82.9	46.2
146.3	77.8	32.8
103.8	59.5	42.1
183.0	68.4	23.7
133.9	124.2	42.1
187.0	87.8	45.2
142.2	94.1	62.1
169.5	69.3	37.8
132.9	66.1	35.3
81.1	52.4	46.3
123.2	75.7	52.2
135.4	72.7	50.5
94.1	77.0	38.3
137.0	78.5	48.4
114.9	78.6	38.8
99.7	54.1	35.2
157.0	86.3	61.9
134.1	85.4	14.0
79.9	43.8	19.7
118.2	60.0	46.7
126.3	82.4	46.1
127.4	75.4	56.4

115.8	63.4	59.4
108.0	64.3	47.6
145.3	76.2	48.0
95.0	62.0	39.8
143.5	72.6	65.1
131.1	75.6	52.0
181.8	91.1	45.4
